# Risk Factors for Facial Appearance Dissatisfaction Among Orthognathic Patients: Comparing Patients to a Non-Surgical Sample

**DOI:** 10.3389/fpsyg.2019.02775

**Published:** 2019-12-11

**Authors:** Pan Shi, Yufei Huang, Hui Kou, Tao Wang, Hong Chen

**Affiliations:** ^1^Faculty of Psychology, Southwest University, Chongqing, China; ^2^School of Management, Zunyi Medical University, Guizhou, China; ^3^Department of Oral and Maxillofacial Surgery, College of Stomatology, Chongqing Medical University, Chongqing, China; ^4^Chongqing Key Laboratory of Oral Diseases and Biomedical Sciences, Chongqing, China; ^5^Chongqing Municipal Key Laboratory of Oral Biomedical Engineering of Higher Education, Chongqing, China; ^6^Key Laboratory of Cognition and Personality, Southwest University, Ministry of Education, Chongqing, China

**Keywords:** facial appearance dissatisfaction, orthognathic patients, media pressure, interpersonal pressure, fear of negative appearance evaluation

## Abstract

This study conducted a cross-sectional investigation of facial appearance dissatisfaction between patients before undergoing orthognathic surgery and a non-surgical sample to evaluate the potential influencing factors of facial appearance dissatisfaction. A sample of 354 participants completed a set of questionnaires concerning facial appearance dissatisfaction, interpersonal pressure, media pressure, and fear of negative appearance evaluation (112 patients, 242 controls). The patients reported higher facial appearance dissatisfaction, more media pressure, more interpersonal pressure, and a greater fear of negative appearance evaluation among others than the control group. Moreover, regression analyses identified interpersonal pressure and fear of negative appearance evaluation as the main predictors of facial appearance dissatisfaction whether in the orthognathic patients or the control groups. The associations between the perceptions of interpersonal pressure, fear of negative appearance evaluation, and facial appearance dissatisfaction support the possible utility of strengthening social experiences and psychological intervention in preventing and treating these appearance-concerns, especially for the orthognathic patients.

## Introduction

Individuals’ satisfaction with their appearance is of central importance to their psychological well-being and health. Previous research has demonstrated negative associations between appearance satisfaction and depression, social avoidance, and low quality of life, both within the general population (Wilson et al., 2013; Griffiths et al., 2017) and among clinical samples with dentofacial deformities who are thought to be particularly vulnerable to dissatisfaction with their facial appearance (Frejman et al., 2013; Feragen and Stock, 2016, 2017). Epidemiological surveys show that about 40% of the population has malocclusion, about 5% of which is due to abnormal skeletal malformation caused by abnormal jaw development, i.e., dentofacial deformity (Qiu, 2008). Dentofacial deformities are changes or irregularities that primarily affect the jaws and teeth. However, multiple craniofacial structures may also be affected (Ong, 2004). In most cases, they are the result of moderate or severe genetic distortions that occurred during the normal development process (such as mandibular prognathism, bimaxillary prognathism or retrognathism, and maxillary vertical excess) and can be corrected using an integrated treatment of orthodontics and orthognathic surgery in adults (Leite et al., 2004).

Facial attractiveness is highly valued in modern society and plays a central role in social activities, such as choosing a partner (Morgan and Kisley, 2014). However, dentofacial deformities cause facial attractiveness to deviate from the sociocultural norms of beauty and attractiveness. This often causes people with dentofacial deformities to feel that they are different from their peers in terms of facial appearance and to suffer from facial appearance dissatisfaction, which may lead to other psychosocial problems such as feelings of hopelessness, interpersonal problems, and low self-esteem (Cadogan and Bennun, 2011; Alanko et al., 2014; Gomes et al., 2019). It has been reported that orthognathic patients have a better personal facial appearance evaluation after orthognathic surgery than before, and their social functioning and self-confidence have improved. These patients also exhibit positive life changes and reduced anxiety (Soh and Narayanan, 2014; Al-Asfour et al., 2018). Although satisfaction with orthognathic treatment is generally high, a small but important group of patients is dissatisfied with the outcome, often despite technically good results (Lee et al., 2007; Ryan et al., 2012). This phenomenon may be caused by psychological factors. Therefore, it is important to investigate psychological factors that may affect patients’ satisfaction with treatment outcomes before offering orthognathic surgery treatment. Therefore, knowledge concerning the specific factors thought to affect the development of subjective facial appearance evaluations among orthognathic patients is essential.

Mass media represents a powerful source of perceived societal values and standards, especially concerning “ideal beauty,” weight, food, and fashion (Levine and Harrison, 2009). An experimental study by Newton and Minhas (2005) demonstrated that female orthognathic patients display more dissatisfaction with their facial appearance after viewing idealized images of facial photographs than females who were not orthognathic patients. Some studies have illustrated that patients with dentofacial deformities perceived social pressure which is created by the media and its representation of “ideal beauty,” but none of these studies have specifically addressed the impact of this pressure on the facial appearance satisfaction of orthognathic patients. Moreover, in the research field of body dissatisfaction, a large number of studies have shown that perceived media pressure can exacerbate an individual’s body dissatisfaction (Robinson et al., 2017; Want and Saiphoo, 2017). Therefore, it is important to discover what the impact of perceived mass media pressure on orthognathic patients’ facial appearance dissatisfaction is.

In addition to mass media, researchers have theorized that messages about appearance-related standards of beauty can also be transmitted by modeling and appearance-related feedback from family members, friends, and romantic partners (Schaefer and Salafia, 2014; Girard et al., 2018). In the field of body dissatisfaction, these messages are often transmitted covertly (e.g., a mother may project a certain message to her daughter about body weight and shape through her dieting habits) or overtly (e.g., a sibling calling their sibling “fat”). Both these messages increase interpersonal pressure for individuals to change their appearance (Chng and Fassnacht, 2016; Thogersen-Ntoumani et al., 2016). This illustrates how weight-related pressure and criticism serves to promote adherence to societal norms concerning weight and shape, further contributing to the individual’s body dissatisfaction. Similarly, associations between interpersonal experiences and subjective appearance evaluations have been described in numerous cross-sectional and quantitative studies within the cleft lip and/or palate (CL/P) literature (Berger and Dalton, 2011; Searle et al., 2017). However, there are no studies comparing the perceived interpersonal pressure regarding facial appearance for people with and without dentofacial deformities. It is reasonable to speculate that due to their unusual facial appearance, orthognathic patients will perceive more interpersonal pressure than those without dentofacial deformities. Furthermore, interpersonal pressure can predict one’s facial appearance dissatisfaction.

Generally, fear of negative evaluation (FNE) can be understood as a type of social anxiety or apprehension about being negatively judged by others and avoidance of situations in which these negative evaluations may occur (Watson and Friend, 1969). Few studies have found a negative correlation between FNE and satisfaction with the appearance in adults with a congenital craniofacial anomaly. Adults with a congenital craniofacial anomaly were more dissatisfied with their facial appearance when they reported an increased FNE (Versnel et al., 2010; Roberts and Mathias, 2013). Importantly, fear of negative appearance evaluation, a domain-specific FNE—which is defined as social anxiety and distress based on having one’s appearance negatively evaluated (Hart et al., 2008)—may also be associated with facial appearance dissatisfaction. Claes et al. (2012) found that fear of negative appearance evaluation was positively correlated with body dissatisfaction in women diagnosed with an eating disorder. Besides, Levinson and Rodebaugh (2012) found that fear of negative appearance evaluation could predict an individual’s body dissatisfaction. However, no research has yet focused on the impact of fear of negative appearance evaluation on facial appearance dissatisfaction.

In summary, psychological factors may play an important role in orthognathic patients’ perception of their facial appearance. According to previous studies on body dissatisfaction, media pressure, interpersonal pressure, and fear of negative appearance evaluation may be the three psychological factors most likely to affect the facial appearance dissatisfaction of clinical patients with dentofacial deformities. However, the first limitation of current research is that no study has focused on the differences between orthognathic patients and a non-surgical sample without dentofacial deformities in facial appearance dissatisfaction, media pressure, interpersonal pressure, and fear of negative appearance evaluation. Moreover, there is limited research that has focused on the role of psychological influences on individuals’ perception of facial appearance to seek better psychological interventions for improving orthognathic patients’ satisfaction with orthognathic surgery treatment outcomes. The current study thus aimed to compare orthognathic patients (patients before undergoing orthognathic surgery) and a non-surgical sample (the controls) in the levels of facial appearance dissatisfaction, appearance pressure from interpersonal relationships and mass media, and negative appearance evaluation fears, as well as to investigate the predictors of facial appearance dissatisfaction among the two groups. In line with previous findings, we hypothesized that (1) the patients would have a higher level of facial appearance dissatisfaction than the controls. Furthermore, relative to the control sample, patients were expected to report more appearance pressure from interpersonal relationships and mass media, and greater negative appearance evaluation fears. We also hypothesized that (2) among the patients and control groups, media pressure, interpersonal pressure, and fear of negative appearance evaluation would be predictors of facial appearance dissatisfaction, and the predictive effect was more significant in patients’ facial appearance dissatisfaction.

## Methods

### Participants

The initial sample consisted of 381 participants (136 patients and 245 controls). Twenty-seven participants (7.09%) were excluded from the sample for being younger than 18 years of age. The final sample consisted of 354 participants [patients: 112 (31.64%), controls: 242 (68.36%)], ranging from 18 to 35 years of age (*M* = 20.78 years, SD = 3.04 years). The patient sample included 112 individuals (females: 76 males: 36) and their ages ranged from 18 to 35 (*M* = 22.79 years, SD = 4.20 years). The patients’ inclusion criteria were: adult patients (more than 18 years old), the presence of skeletal deformity affecting patients’ appearance, is scheduled to undergo orthognathic surgery. The patients’ exclusion criteria were: trauma or previous orthognathic surgery, syndromic patients, cleft lip and palate patients, distraction osteogenesis cases, and patients who had undergone isolated genioplasty procedures. The control group consisted of 242 college students (females: 163, males: 79) who did not have any facial skeletal discrepancies. Additionally, these individuals were not orthodontic patients. Their ages ranged from 18 to 27 years (*M* = 19.85, SD = 1.63). All the participants were from mainland China. Similar to the ethnic group composition of the general population, the great majority of the participants (80.20%) was Han (national minorities: 19.80%). There were no gender differences between the patients and controls, *χ*^2^ (1) = 0.01, *p* = 0.93. Furthermore, there was a significant difference in age between the two groups; *t*(155) = 7.14, *p* < 0.001.

### Procedure

This study was approved by the ethics committee for human research at Southwest University. A doctor’s assistant from the Stomatological Hospital of Chongqing Medical University was instructed thoroughly concerning the study and contacted the orthognathic patients from the Stomatological Hospital of Chongqing Medical University. The patients would receive a survey packet that included a brief research overview, an informed consent agreement, and self-report measures described below. The patients were asked to complete a set of questionnaires carefully. The patient completed the questionnaire 2 days before the surgery. The controls were college students from Zunyi Medical College who did not undergo orthognathic surgery, completed the questionnaires in school hours. This study was carried out from December 2015 to February 2017.

All measures and questionnaires of this study were in Chinese. Several scales (the Sociocultural Attitudes Toward Appearance Scale-3, the Perceived Sociocultural Pressure Scale, and Fear of Negative Appearance Evaluation) were translated into Chinese and back-translated into English in previously published works (e.g., Jackson and Chen, 2010, 2011; Chen and Jackson, 2012). The Negative Physical Self-Face Scale has had a Chinese vision (see Chen et al., 2006).

### Measures

#### Demographic Variables

Participants were asked to provide their age, gender, income levels, relationship status, and nation information.

#### Facial Appearance Dissatisfaction

Facial appearance dissatisfaction was assessed using the Facial Appearance Concern subscale of the Negative Physical Self Scale (NPSS; Chinese version: Chen et al., 2006; Chen and Jackson, 2007). The Facial Appearance Concern subscale consists of 11 items and is comprised of three sub-dimensions: cognition-affect (e.g., “I am depressed about how my face looks”), behavior (e.g., “If it is possible, I will change the way my face looks”), and projection (e.g., “People around me do not like the way my face looks”). All items were rated on a 5-point scale ranging from 0 (never) to 4 (always). Items were averaged to form a total score, with higher scores indicating more facial appearance dissatisfaction. The Facial Appearance Concern subscale has a stable factor structure as well as good reliability and validity among Chinese samples (Chen et al., 2006; Jackson and Chen, 2008; Wang et al., 2019). For the current study, the Cronbach’s alpha values were 0.91 among the patients and 0.90 among the controls.

#### Media Pressure

Reported appearance pressure from mass media was calculated by adding the eight items from the Pressure subscale of the Sociocultural Attitudes Toward Appearance Scale-3 (*SATAQ-3*; Thompson et al., 2004; Chinese version: Chen and Jackson, 2012). Each statement was assessed on a Likert-type scale that ranged from 1 (*totally disagree*) to 5 (*totally agree*). An example item is “I have felt pressure from TV or magazines to change my appearance.” However, four items about thinness (e.g., “I have felt pressure from TV and magazines to be thin”) were removed, as they are not relevant to the research theme. The final score was calculated by the sum of the responses, with higher scores indicating more media pressure. The subscale has satisfactory reliability and validity in Chinese samples (Chen and Jackson, 2012). The Cronbach’s alpha values of the 4-item version of the subscale for the present study sample were 0.91 among patients and 0.81 among the controls.

#### Interpersonal Pressure

Six items of the Perceived Sociocultural Pressure Scale-Interpersonal (*PSPS-I*; Stice and Agras, 1998; Chinese version: Jackson and Chen, 2008) were used to assess appearance pressure from friends (e.g., “I have felt pressure from my friends to change my physical appearance”), family (e.g., “I have felt pressure from my family to change my physical appearance”) and prospective romantic partner (“I have felt pressure from people that I am interested in dating to change my physical appearance”). Each item was rated from 1 (*none*) to 5 (*a lot*). For the Chinese samples, items have been modified slightly to reflect pressure to “change my physical appearance” or “have a certain physical appearance” rather than pressure to be thin (e.g., Jackson and Chen, 2008). Concerning the “romantic partner” items, these were changed to include the respondent’s “current romantic partner or people I would like to date” to increase applicability to all respondents, including those not currently dating. The final score was calculated by the sum of the responses, with higher scores indicating more interpersonal pressure. Chen and Jackson (2012) found all six interpersonal PSPS items loaded on one component in Chinese samples of each gender. The Cronbach’s alpha values for this variable were 0.86 for patients and 0.79 for the controls.

#### Fear of Negative Appearance Evaluation

The six-item fear of negative appearance evaluation (*FNAE*, Lundgren et al., 2004; Chinese version: Jackson and Chen, 2011) assessed the frequency of concerns that others will judge one’s physical appearance negatively. Sample items included “I worry that people will find fault with the way I look” and “I am afraid other people will notice my physical flaws.” Each item was rated from 1 (*never*) to 5 (*almost always true*). The final score was calculated by the sum of the responses, with higher scores indicating a greater FNAE. The questionnaire showed good reliability for Chinese adolescents (Jackson and Chen, 2011). The Cronbach’s alpha values were 0.90 for patients and 0.84 for controls.

### Statistical Analyses

The statistical analyses were performed using SPSS 22. Descriptive analyses were conducted to obtain the ranges, means, and standard deviations of the two study groups (Table 1). Next, we performed a multivariate analysis of covariance (MANCOVA) controlling for age (covariates) between the patients and the controls for there was a significant difference in age between the two groups. To explore the relationships among facial appearance dissatisfaction, and risk factors (media pressure, interpersonal pressure, and fear of negative appearance evaluation), the correlations among the variables were initially examined in patients and controls separately by using a Pearson correlation (Table 2). We used a recommended guideline by Gignac and Szodorai (2016) for interpreting the magnitude of correlations (0.15 = small, 0.25 = medium, and 0.35 = large). Hierarchical multiple regression analyses were also conducted to determine the contribution of the variables of interest on facial appearance dissatisfaction, controlling the model with the age and gender of the patients and controls separately (Table 3). Multicollinearity was examined using tolerance and VIF statistics and found to be acceptable in all cases. Highest VIF values were 2.26, and lowest tolerance values were 0.44.

**Table 1:** Scale range, mean, and standard deviation (SD) for study variables among orthognathic patients and the control group.

	Participants (*N* = 354)				*F* *df* (1, 350)	*η*^2^
Measures	Patients (*N* = 112)		Controls (*N* = 242)			
	Range	*M ±* SD	Range	*M ±* SD		
Facial appearance dissatisfaction	0.27–3.73	1.60 ± 0.73	0–3.73	1.08 ± 0.70	32.83^***^	0.09
Media pressure	4–20	10.29 ± 3.99	4–20	8.89 ± 3.39	9.66^**^	0.03
Interpersonal pressure	6–26	13.64 ± 4.81	6–27	11.91 ± 4.23	8.34^**^	0.02
Fear of negative appearance Evaluation	7–30	17.99 ± 5.38	6–30	14.91 ± 4.65	26.10^***^	0.07

** p < 0.01;

*** *p < 0.001*.

**Table 2:** Correlations between studied variables for orthognathic patients and the control group.

Variables	1	2	3	4	5	6
1. Age	—	—	−0.02	0.01	−0.04	−0.05
2. Gender	—	—	0.03	−0.02	0.07	−0.06
3. Facial appearance dissatisfaction	**0.12**	**0.02**	**—**	0.39^**^	0.43^**^	0.60^**^
4. Media pressure	**−0.02**	**−0.06**	0.44^**^	**—**	0.65^**^	0.51^**^
5. Interpersonal pressure	**0.06**	**−0.01**	0.52^**^	0.69^**^	—	0.45^**^
6. Fear of negative appearance evaluation	**−0.01**	**−0.002**	0.67^**^	0.57^**^	0.53^**^	**—**

** *p < 0.01*.

*Values for orthognathic patients are in **bold**; values for the controls are in not bold*.

**Table 3:** Hierarchical multiple regression analyses of risk factors predicting facial appearance dissatisfaction for orthognathic patients and the control group.

Predictors	Patients			Controls		
	SE	*β*	*t*	SE	*β*	*t*
**Step1**	*F*_change_(2,109) = 0.08 *R*^2^_change_ = 0.001			*F*_change_(2,239) = 0.15 *R*^2^_change_ = 0.001		
Age	0.07	0.01	0.20	0.52	0.01	0.10
Gender	0.15	0.02	0.35	0.11	0.04	0.83
**Step2**	*F*_change_(3,106) = 34.78 *R*^2^_change_ = 0.50^***^			*F*_change_(3,236) = 49.97 *R*^2^_change_ = 0.39^***^		
Media pressure	0.10	−0.11	−1.01	0.07	0.01	0.12
Interpersonal pressure	0.10	0.29	2.91^**^	0.07	0.19	2.69^**^
Fear of negative appearance evaluation	0.09	0.58	6.65^***^	0.06	0.51	8.50^***^

*All of the studied variables were mean-centered except for gender*.

** p < 0.01;

*** *p < 0.001*.

## Results

### Between-Group Differences in Facial Appearance Dissatisfaction, Risk Factors

Table 1 presents the scale range, mean values, and standard deviations (SD) of the study variables among the orthognathic patients and the control group.

In accordance with our first research question, the multivariate analysis of covariance revealed significant differences in the combined variables, *F* (4, 347) = 9.13, *p* < 0.001, *η*^2^ = 0.10; Pillai’s trace = 0.10. An examination of the univariate values of *F* indicated that compared to the control group, the patients reported higher facial appearance dissatisfaction, more media pressure, more interpersonal pressure, and a greater fear of negative appearance evaluation among others (Table 1).

### Predictors of Facial Appearance Dissatisfaction

The bivariate correlations among all the study variables for both samples are presented in Table 2. For both the patients and control groups, the pattern of correlations confirmed the expected relationships between the study variables. Furthermore, the findings concerning the bivariate correlations reveal that in both samples, facial appearance dissatisfaction has a strong association with media pressure (patients: *r* = 0.44, *p* < 0.01; controls: *r* = 0.39, *p* < 0.01), interpersonal pressure (patients: *r* = 0.52, *p* < 0.01; controls: *r* = 0.43, *p* < 0.01), and fear of negative appearance evaluation (patients: *r* = 0.67, *p* < 0.01; controls: *r* = 0.60, *p* < 0.01).

Starting with these significant associations, we conducted hierarchical multiple regression analyses with facial appearance dissatisfaction as a dependent variable (Table 3). We also proposed independent models for patients and the controls. Because previous analyses revealed age- and gender-related variations in body dissatisfaction (Menzel et al., 2010; Chng and Fassnacht, 2016), to exclude the influence of irrelevant variables in this study, we accounted for the baseline level of these demographic variables in the first step.

For the orthognathic patients, after the age and gender analyses, the predictors explained 50% of the variance in facial appearance dissatisfaction changes (Table 3). The significant predictors were interpersonal pressure (*β* = 0.29, *p* < 0.01) and fear of negative appearance evaluation (*β* = 0.58, *p* < 0.001). Moreover, fear of negative appearance evaluation was the strongest predictor of facial appearance dissatisfaction for orthognathic patients. In this model, media pressure had no significant influence (*p* > 0.05).

Concerning the control group, the hypothesized predictors accounted for 39% of the variance in facial appearance dissatisfaction changes after controlling for age and gender (Table 3). The significant predictors were interpersonal pressure (*β* = 0.19, *p* < 0.01) and fear of negative appearance evaluation (*β* = 0.51, *p* < 0.001). Moreover, fear of negative appearance evaluation was considered to be the strongest predictor of facial appearance dissatisfaction, with media pressure again having no significant effect (*p* > 0.05).

In light of our original research question, these results suggest that while for both groups the results of hierarchical multiple regression analyses are the same, the associations between interpersonal pressure and fear of negative appearance evaluation and facial appearance dissatisfaction are stronger in orthognathic patients than in the control group (see Table 3).

## Discussion

This study is the first to investigate the relationship between psychological factors and facial appearance dissatisfaction when considering orthognathic patients and a non-surgical sample group. First, we determined the differences between the two groups concerning facial appearance dissatisfaction, media pressure, interpersonal pressure, and fear of negative appearance evaluation. Second, the findings contribute to our knowledge of interpersonal pressure and fear of negative appearance evaluation and their relationships with facial appearance dissatisfaction for both orthognathic patients and the control group.

The findings revealed that the orthognathic patients felt more dissatisfied with their facial appearance than the comparison groups. This is consistent with earlier research on the topic which has found patients who pursue orthognathic surgery were more concerned about their dentofacial appearance and general body image compared with the individuals who did not have skeletal discrepancies (Sar et al., 2015). Furthermore, the patients perceived more media pressure, interpersonal pressure, and reported greater fear of negative appearance evaluation than the controls. These findings are consistent with the patients’ situation before orthognathic surgery. The patients feel that their dentofacial deformities make them look different from the peers and reduce their facial attractiveness, affecting many aspects of their lives, such as social interactions, their possible success when seeking employment, being chosen as a romantic partner, and not least, their personality and characteristics (Eslamipour et al., 2017). These differences between patients and the controls indicate that it is essential to investigate the risk factors concerning patients’ facial appearance dissatisfaction and the related psychosocial factors, rather than only investigating the biological influence.

The predictive effects of the risk factors concerning facial appearance dissatisfaction in patients and controls were examined separately and the findings partially answered our research question. Generally, the patients and the controls showed the same pattern of results.

For both groups, interpersonal pressure reliably predicted concerns regarding facial appearance, suggesting that interpersonal pressure may have a negative influence on an individual’s facial appearance satisfaction. This finding is consistent with similar research in body dissatisfaction literature, which presented that direct interpersonal influences can predict body dissatisfaction (Johnson et al., 2015). The interpersonal pressure may come from daily life communication overtly (e.g., appearance teasing from peers, parental comments regarding facial appearance) or covertly (e.g., parents worry that their children are disadvantaged in society because of facial appearance), and these pressures may profoundly affect the development and construction of individuals’ facial appearance dissatisfaction.

The expected relationship between fear of negative appearance evaluation and facial appearance dissatisfaction was confirmed for both patients and controls. The result showed that fear of negative appearance evaluation was the most predictive variable on facial appearance dissatisfaction, highlighting the centrality of this variable for facial appearance dissatisfaction. The observations in this study are consistent with that of Lundgren et al. (2004), who found that appearance evaluation fears predicted body image concerns. Similarly, Jackson and Chen (2011) found that fear of negative appearance evaluation was among the strongest predictors of changes in eating pathology for Chinese girls. Our results suggest that elevated fear of negative appearance evaluation may be a risk factor for the development of facial appearance dissatisfaction.

The lack of significance of the media in predicting facial appearance dissatisfaction among both patients and the non-surgical sample in this study is also of interest. There was a positive correlation between media pressure and facial appearance dissatisfaction for both groups. Contrary to our results, there is a substantial body of research that indicates that the media was the main source of influence on body dissatisfaction (Robinson et al., 2017; Want and Saiphoo, 2017). The sample size of orthognathic patients and non-clinical samples in this study is small, which may affect the statistical significance of media pressure. Another possible explanation is the difference in the findings may relate to mass media playing an important role in conveying body shape and weight ideals, rather than specifically facial features (e.g., Thompson et al., 1999; Thompson and Smolak, 2001). Contrary to body shape and weight ideals, there is no “gold standard” for facial beauty for the perceptions vary among various people (Rhodes, 2006), which may weaken the effect of media pressure. However, on the other hand, experimental research showed that exposure to idealized images of facial photographs transmitted by mass media can increase facial appearance dissatisfaction in female orthognathic patients (Newton and Minhas, 2005). There may be some discrepancies between the media pressure of self-report and the pressure caused by direct media exposure.

### Limitations and Future Research Directions

A particular strength of this research is that we extended our research beyond body dimensions to facial characteristics to investigate the risk factors of facial appearance dissatisfaction among orthognathic patients and the controls. However, several limitations also need to be recognized. The first of these limitations is that it is not possible to conclude the direction of the relationship between the factors investigated due to the cross-sectional design of the study. Further clarification of these relationships requires studies using experimental or longitudinal designs. Second, a mismatch of the number of patients and the controls existed. Additional prospective research with larger clinical samples may be warranted. Third, this study lacks the representativeness of the clinical sample. It is difficult to apply the results to other clinical groups who typically undergo cosmetic surgery to alter specific facial features, such as “double-eyelids” and large eyes, a melon-shaped face, and small, proportionate, defined chin. Fourth, gender differences were found concerning shame regarding one’s body and shape/weight concerns, revealing that women experience more body dissatisfaction than do men (Else-Quest et al., 2012; Buchanan et al., 2013). Lastly, we did not collect the educational level of all the subjects, which may affect the differences in the psychological factors between the two groups.

Although this study found the predictive effect of interpersonal pressure on facial appearance dissatisfaction, most existing studies focused on the impact of the specific aspects of interpersonal pressure on body dissatisfaction and have found evidence concerning the impact of different forms of interpersonal pressure, such as peer pressure and parental pressure (Helfert and Warschburger, 2011; Vries et al., 2015; Girard et al., 2018). Future research is needed to examine the effects of interpersonal pressure from family members, friends, and romantic partners on facial appearance dissatisfaction, and to clarify how an individual who is dissatisfied with oneself appearance to interpret and internalize these experiences. Moreover, as mentioned above, the media pressure of self-report and the pressure caused by direct media exposure on facial appearance dissatisfaction may have a different role. Future research is required to further clarify the precise mechanisms of media message to facial appearance dissatisfaction.

### Clinical Implications

First, there were differences between patients and the controls concerning facial appearance dissatisfaction, media pressure, interpersonal pressure, and fear of negative appearance evaluation. These differences between the two groups indicate that the psychosocial factors for orthognathic patients are needed to be paid attention to before the surgical treatment.

Second, we found that facial appearance dissatisfaction was predicted by interpersonal pressure and fear of negative appearance evaluation in both orthognathic patients and non-patients groups. Furthermore, interpersonal pressure and fear of negative appearance evaluation were more strongly associated with facial appearance dissatisfaction for the orthognathic patients. These findings further stress the importance of an interdisciplinary team in building the doctor team, including psychologists when working with people with dentofacial deformities. Psychologists should use measurement to assess the orthognathic patients’ psychological state, and use reasonable interventions to adjust the psychological problems caused by the disease. More specifically, given that the fear of negative appearance evaluation has the strongest influence for orthognathic patients in this study, the psychologists in the team should pay attention to the role of fear of negative appearance evaluations and work toward changing people’s attitudes concerning the negative appearance feedback to improve their facial appearance satisfaction. We believe that the research presented here is a step toward understanding negative appearance evaluation fears as an important risk factor for facial appearance dissatisfaction. Future research can further explore the psychological interventions used to change the fear of negative facial appearance in patients with dentofacial deformities.

## Conclusion

In conclusion, the findings of this study expand upon previous work in the field by exploring the effects of risk factors of facial appearance dissatisfaction in both orthognathic patients and non-surgical controls. Generally, the patients and the controls showed the same pattern of results. The results suggest that interpersonal pressure and fear of negative appearance evaluation play a crucial role in facial appearance dissatisfaction for orthognathic patients as well as the non-surgical sample. More significantly, the findings point to the possibility that interventions promoting facial appearance satisfaction could change people’s attitudes toward appearance feedback from friends or family, especially for orthognathic patients. In building the doctor team, it is important to form an interdisciplinary team, including psychologists when working with people with dentofacial deformities. Although the focus of orthognathic surgery procedures is on correcting the morphological deformity, the assessment and treatment plan should also involve the psychosocial aspects of orthognathic patients.

## Data Availability Statement

The datasets generated for this study are available on request to the corresponding author.

## Ethics Statement

The studies involving human participants were reviewed and approved by the Southwest University Human Ethics Committee. The patients/participants provided their written informed consent to participate in this study.

## Author Contributions

PS made substantial contributions to the interpretation of data. PS and YH designed the study and wrote the protocol. HK was responsible for data collection. TW revised the language. HC conducted literature searches and provided summaries of previous research studies. All authors gave final approval of the version to be published and agreed to be accountable for all aspects of the work.

### Conflict of Interest

The authors declare that the research was conducted in the absence of any commercial or financial relationships that could be construed as a potential conflict of interest.
